# Failure to correct the international normalized ratio in patients with anticoagulant-related major bleeding is associated with increased 90-day mortality

**DOI:** 10.1186/cc9852

**Published:** 2011-03-11

**Authors:** J Menzin, L White, M Friedman, C Nichols, JA Menzin, C Jones, J Hoesche

**Affiliations:** 1 Boston Health Economics, Inc., Waltham, MA, USA; 2 CSL Behring, King of Prussia, PA, USA

## Introduction

Supratherapeutic international normalized ratio (INR) levels have been shown to be a significant predictor of death among patients with anticoagulant-related (ACR) intracranial hemorrhage (ICH). We assessed factors associated with 90-day mortality and time to death in patients receiving fresh frozen plasma (FFP) for ACR major bleeding in clinical practice.

## Methods

A retrospective analysis was undertaken using electronic medical records from an integrated system. Patients who received FFP between 1 January 2004 and 31 December 2010, and who met the following criteria were selected: major hemorrhage diagnosis the day before to the day after initial FFP administration; INR ≥2 on the day before or the day of FFP and another INR result up to 1 day after FFP; and warfarin supply within 90 days prior to hospitalization. INR correction (defined as INR ≤1.3) was evaluated at the last available test up to 1 day following FFP start. Patients dying within 72 hours surrounding FFP were excluded. Kaplan-Meier survival curves were estimated and time to death was assessed using Cox proportional hazards models. In sensitivity analysis, an INR threshold ≤1.5 was used to account for clinical practices that aim to avoid adverse outcomes (for example, thrombosis) of certain co-morbidities.

## Results

A total of 405 patients met the selection criteria (mean age 75 years, 53% male), and 67% remained uncorrected. Overall, 19% of patients died within 90 days of hospital admission, with a higher proportion of uncorrected versus corrected patients dying (24% vs. 13%, *P *= 0.013). In Cox regression analysis, patients with a first elevated INR value >4 (HR = 2.21; 95% CI = 1.36 to 3.60), with an ICH bleed versus gastrointestinal or other bleed (HR = 2.08; 95% CI = 1.27 to 3.40), and with uncorrected INR (HR = 2.33; 95% CI = 1.30 to 4.16) were significantly more likely to die within 90 days of admission. In a sensitivity analysis (correction defined as INR ≤1.5), 39% remained uncorrected within 24 hours of FFP administration, with factors predicting 90-day mortality remaining robust in regression analysis.

## Conclusions

Among ACR major bleed patients, not correcting to either INR ≤1.3 or INR ≤1.5 with FFP is associated with an increased rate of mortality at 90 days. Further assessment of co-morbidities associated with hemostasis and other predictors of mortality risk in this population is warranted.

